# The Impact of Nanomedicine on Soft Tissue Sarcoma Treated by Radiotherapy and/or Hyperthermia: A Review

**DOI:** 10.3390/cancers16020393

**Published:** 2024-01-17

**Authors:** Maria-Eleni Zachou, Vassilis Kouloulias, Marina Chalkia, Efstathios Efstathopoulos, Kalliopi Platoni

**Affiliations:** 2nd Department of Radiology, Medical School, Attikon University Hospital, National and Kapodistrian University of Athens, 11527 Athens, Greece; vkouloul@med.uoa.gr (V.K.); chalkiamar@outlook.com (M.C.); stathise@med.uoa.gr (E.E.)

**Keywords:** soft tissue sarcoma, nanoparticles, radiotherapy, hyperthermia

## Abstract

**Simple Summary:**

Our manuscript addresses a noteworthy gap in the cancer research literature through an in-depth exploration of existing studies on the potential application of nanoparticles in radiotherapy and/or hyperthermia treatment for soft tissue sarcomas (STS). While nanoparticles have shown promising results in various cancer types, STS, known for its radioresistant and aggressive nature, lacks comprehensive research. Our aim is to analyze and compare the current body of literature, in order to provide a new perspective on the use of nanoparticles in enhancing radiotherapy and hyperthermia treatment outcomes for STS. The significance of our work lies in its potential to consolidate knowledge, identify research gaps, and offer insights into nanoparticle-based therapies for STS. By shedding light on existing studies, our literature review provides valuable information for the research community, fostering a deeper understanding of potential strategies for improving therapeutic approaches to this challenging form of cancer.

**Abstract:**

This article presents a comprehensive review of nanoparticle-assisted treatment approaches for soft tissue sarcoma (STS). STS, a heterogeneous group of mesenchymal-origin tumors with aggressive behavior and low overall survival rates, necessitates the exploration of innovative therapeutic interventions. In contrast to conventional treatments like surgery, radiotherapy (RT), hyperthermia (HT), and chemotherapy, nanomedicine offers promising advancements in STS management. This review focuses on recent research in nanoparticle applications, including their role in enhancing RT and HT efficacy through improved drug delivery systems, novel radiosensitizers, and imaging agents. Reviewing the current state of nanoparticle-assisted therapies, this paper sheds light on their potential to revolutionize soft tissue sarcoma treatment and improve patient therapy outcomes.

## 1. Introduction

In recent years, nanotechnology has emerged as a dominant and rapidly developing topic across various scientific and engineering disciplines. Its profound impact on healthcare has led to the evolution of nanomedicine, where nanotechnology is seamlessly integrated into medical practices, giving rise to innovative approaches to treatments, prosthetics, and diagnostics [1]. Nano-drugs, typically ranging from 1 to 100 nm in size with an unrestricted upper limit, possess exceptional optical, electrical, physical, and chemical properties, due to their increased surface area to volume ratio [2]. As a result, nanomedicine aims to address unresolved issues and diseases, including autoimmune disorders, cardiovascular ailments, and notably cancer.

Traditionally, cancer treatment has relied on surgery, RT, and chemotherapy. While these methods can effectively target cancer cells, their associated toxicities often limit treatment success. Systemically administered chemotherapy drugs lead to cytotoxic effects throughout the body, causing severe side effects beyond the tumor site [3]. In the case of RT, the challenge lies in sparing surrounding healthy tissues from radiation damage, while delivering sufficient doses to the tumor [4]. Additionally, HT as a cancer treatment requires precise temperature control to avoid under-treatment or thermal ablation [5].

To overcome the limitations of existing cancer treatment methods, researchers have begun exploring the integration of nanoparticles (NPs) in various therapeutic approaches. In chemotherapy, liposomal drug delivery agents, such as liposomal doxorubicin (DoxilR), have already found clinical application. Efforts are underway to enhance specific targeting by functionalizing NP surfaces with cancer-specific antibodies and achieving better control over drug release rates [6]. Similarly, in RT, nanomedicine is focused on developing radio-sensitizing agents, specifically high-atomic number NPs, which have shown promising radio-sensitizing effects in various cancer types [7]. Furthermore, NPs have been implemented in HT, either for intrinsic temperature induction using metallic or magnetic NPs with alternating electromagnetic fields or for the targeted release of chemo-drugs from thermally responsive NPs with external sources [8,9].

These advancements in nanomedicine have yielded promising results in preclinical and clinical trials of chemotherapy [10,11], RT [12,13], and HT [14,15], opening up new possibilities for cancer diagnosis and treatment. NP-based strategies can also be applied for simultaneous imaging with MRI [16] or CT [17], offering dual functionality. Given the multiple combinations of NP applications, the future holds great promise for the fight against cancer.

In RT cancer treatment, NPs are mainly implemented as radiosensitizers. The hypoxic environment within tumors poses a significant challenge to the success of RT treatment, as the indirect effect of radiation, e.g., the production of reactive oxygen species (ROS) that chemically affect the DNA of the cells and cause cellular death, will not be as effective. To overcome this limitation, the development of radiosensitizing agents has been pursued. In the 1980s, the concept of using high-Z (atomic number) materials was introduced. When high-Z atoms interact with photon radiation, they cause strong attenuation, resulting in an increased dose deposition in the localized area where they are present [18].

Clinical RT commonly employs photon energies ranging from 6 MV to 25 MV. Within this energy range, the primary interaction between the radiation beam and soft tissue is the Compton effect. Notably, the Compton effect remains unaffected by the administration of high-Z agents, since it is independent of the Z-number of the material. On the other hand, the photoelectric effect is highly influenced by the Z-number (~ $Z^{3}$ ) of the material. By introducing a high-Z material into the treated area, there is an increase in photoelectric interactions, leading to the production of more photoelectrons and secondary photons. Additionally, this results in the release of Auger electrons with a high linear energy transfer (LET). Consequently, more ionizations occur in the region, causing enhanced cellular damage through oxidative-stress mechanisms [7]. These materials are called nano-enhancers and include bismuth (Z = 83), gold (Z = 79), tungsten (Z = 74), tantalum (Z = 73), hafnium (Z = 72), and silver (Z = 47).

HT, the controlled application of mild heat (typically 41–45 °C) for therapeutic purposes, has a rich historical background in cancer treatment. Initially used in ancient times, it regained medical interest in the 20th century and is now considered a fourth mainstay in cancer treatment alongside surgery, chemotherapy, and RT. HT is classified into local, regional, and whole-body approaches, each tailored to specific cancer scenarios. Techniques like microwave probes, radiofrequency probes, laser, ultrasound, heating fluids, and hot water baths are used for heat induction [5,19].

The impact of HT on cells is multifaceted, affecting proteins, nucleic acids, and cellular cycles. It can induce apoptosis and activate the immune system by displaying tumor-specific antigens on cell surfaces. Moreover, HT’s synergistic effects with RT and chemotherapy are valuable, enhancing tumor oxygenation, blood circulation, and sensitizing tumors to radiation, while not relying on cell cycle phases for efficacy. This combined approach offers promising benefits in cancer treatment and continues to be a subject of research and development, while nowadays HT is included in clinical practice for several solid tumors [20,21,22].

Despite the considerable progress in applying NPs to cancer treatments, their integration into the clinical practice of RT and HT for STS remains limited. STS, which is an aggressive cancer type prevalent among younger individuals, poses challenges for treatment, due to a poor prognosis and the potential for severe side effects associated with conventional therapies, including radiation-induced secondary cancers and the need for amputations [23].

RT for STS is delivered either preoperatively or postoperatively. The study of O’Sullivan et al. [24], exploring the role of preoperative vs. postoperative radiotherapy in soft tissue sarcomas, reported that wound complications were 35% vs. 17%, respectively (*p* = 0.01), while overall survival was better in the preoperative group (*p* = 0.048). The authors concluded that choice of treatment for patients with soft tissue sarcoma should take into account the timing of surgery and radiotherapy, and the size and anatomical site of the tumor. However, in another randomized study by Davis et al. [25], timing of RT in terms of pre vs. post-operative setting, had no significant impact on the function of STS patients in the first year after surgery. Moreover, in a later study by Davis et al. [26], fibrosis was greater in the postoperative compared to preoperative period (*p* = 0.07), while filed size was also a relevant significant factor. As a matter of fact, the role of the timing of RT before or after surgery remains unclear, while the preoperative setting seems to have trend of better OS and less fibrosis. Since the preoperative setting might be a better choice, NPs would offer an enhancement in RT intratumoral without increasing the surrounding fibrosis.

The existing literature on the use of NPs for STS primarily consists of preclinical studies on STS cell lines or animal models, with only a few phase I and phase II/III clinical trials [27,28] and a limited number of veterinary studies [29,30,31]. In this context, this paper aims to comprehensively review the published literature on the application of NPs in the treatment of STS with RT and HT, as no such research has been published so far. An overview of the existing knowledge, potential benefits, limitations, and areas where further research is needed is provided.

## 2. Materials and Methods

The present study is a comprehensive review of published studies, gathered from several online sources. The primary research tool utilized was PubMed, using specific keywords with the PubMed Advanced Search Builder. The search terms included “Soft tissue sarcoma”, “Soft tissue sarcoma treatment”, “Soft tissue sarcoma-Radiotherapy”, “Soft tissue sarcoma-radiotherapy-nanoparticles”, “Soft-tissue sarcoma-hyperthermia”, “Soft tissue sarcoma-hyperthermia-nanoparticles”. Additionally, the same keywords were employed for searches in Google Scholar. Complementary research was conducted using the search engine Google.

The initial search with terms such as nanoparticles and radiotherapy, and nanoparticles and hyperthermia, resulted in numerous results across various cancer types. However, to focus specifically on soft tissue sarcomas, the search was limited to articles pertaining to experimental and clinical studies of this particular cancer type, excluding review articles through the use of filters, and the publication dates were constrained from 2005 to today. This study includes the majority of articles obtained through this targeted approach.

The results for STS NPs and RT were primarily focused on gold nanoparticles, since gold nanoparticles (GNPs) have been thoroughly researched and are well known for their biocompatibility and low systemic toxicity. GNPs have been successfully used as radiosensitizers against various tumors treated with radiotherapy, as their strong photoelectric absorption coefficient leads to an increased local dose deposition. The other major category of NPs described in this study is NBTXR3, as it was the first type to be used for human trials for the treatment of STS. In the case of hyperthermia, the NPs most thoroughly investigated, and hence included in this review, are thermosensitive liposomes and magnetic NPs. HT, has been observed to make blood vessels in tumors more permeable to liposomes. Additionally, HT can be employed as a method to improve the delivery of drugs enclosed within liposomes to tumors, through causing the release of the drug from the liposomes [32].

## 3. Results

### 3.1. Nanoparticles Used in Radiotherapy Treatment of Soft Tissue Sarcomas

#### 3.1.1. Gold Nanoparticles

The effectiveness of radiosensitization with GNPs in STS was clearly observed in a study published in 2013 [33]. The scientific team used pegylated gold nanoparticles (P-GNP) on a HT1080 fibrosarcoma cell line. The GNPs consisted of a 12 nm gold core and conjugated Poly (ethylene glycol) (PEG) chains, reaching a total diameter of approximately 26 nm. The NPs had a low polydispersity index, presenting an increased homogeneity. Their zeta-potential was −0.55 mV, indicating effective shielding. In that study, the radiosensitization was monitored both in vitro and in vivo.

In vitro, when HT1800 cells were exposed to P-GNPs, more unrepaired double strand breaks occurred compared to with radiation alone. Clonogenic survival assays revealed a sensitization enhancement ratio (SER) of 1.16 at a 10% survival fraction, suggesting enhanced radiosensitization for this cell line. In vivo tests involved subcutaneously implanting HT1080 cells into athymic nude (nu/nu) mice. Cone beam CT imaging post-injection showed an extended circulation half-life and increased tumor deposition, enabling clear tumor differentiation from healthy tissue. Histological analysis of injected and uninjected mice 48 h post-injection confirmed significantly higher NP accumulation in tumors, particularly near tumor vasculature, thanks to the EPR effect. Monitoring tumor volume and overall survival in RT-treated mice demonstrated greater tumor regression and higher survival rates with P-GNP+RT.

In an alternative approach of NP implementation for contrast enhancement, Miladi et al. integrated GNPs with magnetic resonance contrast agents [34]. Specifically, GNPs were coated with diethylenetriaminepentaacetic acid-gadolinium chelates (Au@DTDTPA-Gd).

Following successful in vitro tests to characterize the NP properties, the same research team implemented in vivo experiments with 9L gliosarcoma (9LGS). Tumors were surgically implanted in mouse right brain hemispheres, and Au@DTDTPA-Gd NPs were intravenously injected. Real-time monitoring via T1-weighted images revealed a crucial signal on the right brain hemisphere, signifying a critical temporal window between the third and seventh minute post-injection, when NP concentration induced notable radiosensitization.

Based on these findings, the team conducted microbeam radiation therapy (MRT) on rats, precisely delivering high doses to the treatment area, while sparing surrounding healthy tissues—crucial for central nervous system (CNS) tumors. Irradiation occurred during the fithf minute post-NP injection, resulting in a remarkable 473.3% increase in the experimental group’s lifespan compared to the control group, highlighting the pronounced radiosensitizing effect.

An intriguing aspect of this study is the NPs’ ability to breach the blood–brain barrier, a significant challenge in drug delivery. Leveraging the enhanced permeability and retention (EPR) effect, these NPs accumulated within the brain tumor, contrary to earlier studies where NPs could not breach the blood–brain barrier. This suggests tailored engineering of GNPs for specific tumor locations and desired therapeutic outcomes. In summary, this study showcased GNPs potential for site-specific therapies, overcoming anatomical barriers for more effective CNS tumor treatment—a remarkable breakthrough in cancer research and treatment.

The mentioned studies aimed to enhance the therapeutic effects of RT in radioresistant STSs, utilizing NPs for both radio enhancement and contrast imaging. In a subsequent study, two different NPs were employed to evaluate the impact of RT alone and RT enhanced with GNPs on tumor vasculature [35]. PEGylated GNPs and RGD peptide-functionalized GNPs, which bind to receptors on tumor neovasculature endothelial cells [36,37], were intravenously administered to rats with primary STS. One day post-injection, the rats were irradiated with 20 Gy using an X-RAD 225 Cx small animal image-guided irradiator (225 kVp). Additionally, one day post-irradiation, rats received liposomal iodine NPs as a contrast agent. Dual-energy CT scans were performed, and dual-energy decomposition was utilized to create concentration maps of gold and iodine within each tumor.

The research findings revealed a notable threshold for vascular permeability. No discernible change in iodine accumulation was observed following irradiation at doses within the 0–10 Gy range. However, a significant increase in accumulation was noted at the 20 Gy level. Remarkably, the same concentration of liposomal iodine within the tumor was achieved with just 5 Gy of radiation when incorporating RGD-GNPs. This highlights the profound radiosensitizing effect of targeted GNPs, leading to substantial disruption of the tumor’s vascular epithelium.

This disruption resulted in increased liposomal doxorubicin concentrations within the tumor, facilitated by the enhanced permeability and retention (EPR) effect. Assessing these findings, the scientific team replaced liposomal iodine with liposomal doxorubicin, to assess the combined effects of RT and chemotherapy. The treatment’s efficacy was evaluated based on the tumor volume doubling time. The combined strategy of using RGD-GNPs to enhance RT and liposomal doxorubicin yielded highly promising results. Notably, it led to a significant difference in tumor doubling time, with the combination therapy achieving more than twice the delay in tumor growth compared to the control groups

#### 3.1.2. Hafnium Oxide Nanoparticles for the Radiotherapy of STS

Hafnium oxide NPs, known as NBTXR3, have undergone clinical testing for RT enhancement of soft tissue sarcomas (STS) [38]. NBTXR3 NPs possess key characteristics that make them promising for therapy. Sized at 50 nm, they enhance biodistribution, exploiting the enhanced permeability and retention (EPR) effect to accumulate efficiently within tumor tissues. Their spherical shape and negative surface charge ensure stability, improve bloodstream circulation, and facilitate cellular uptake [39]. Moreover, their high atomic number (Z = 72) renders NBTXR3 an effective radiosensitizer.

Early in vivo and in vitro experiments with xenografted HT1080 fibrosarcoma tumors in rats demonstrated enhanced radiosensitivity when NBTXR3 was combined with RT, using both Cobalt-60 and a 6 MV linear accelerator beam.

Tests were extended to A673 xenografted tumors, representing a human Ewing family-type sarcoma model. Mice injected with NBTXR3 were irradiated with Cobalt-60 at a 15 Gy dose. The treatment with NPs, assessed through tumor regrowth delay, showed a significant improvement compared to RT alone.

Importantly, NBTXR3 NPs activate only in response to irradiation, causing no toxicity. Their tendency to persist intracellularly makes them suitable for fractionated RT regimens.

Promising results in initial experiments led to a Phase I clinical trial by Bonvalot et al. [27]. Patients diagnosed with various types of STS received NBTXR3 intratumorally, guided by ultrasound. A 50 Gy 3D-RT regimen was administered over 5 weeks, followed by surgery.

The results indicated that the recommended dose (RD) for NBTXR3 treatments should be 53.3 g/L, administered at a volume equivalent to 10% of the baseline tumor volume. Patients treated with this dose experienced a significant reduction in tumor diameter (29%) and volume (40%), facilitating safer surgical procedures.

Promising outcomes prompted a Phase II/III clinical trial with 180 STS patients [28] The evaluation focused on pathological complete response (pCR) and overall antitumor response.

NBTXR3+RT outperformed RT alone, with a pCR rate of 16% vs. 8% in the intention-to-treat group and 19% vs. 9% in the per-protocol group. Histological evaluations revealed the greater efficacy for grade 2 and 3 tumors, highlighting the need for further research on differential tumor grade responses. NBTXR3+RT also resulted in a higher percentage of patients achieving negative resection margins (84% vs. 70% with RT alone), associated with lower tumor recurrence risk.

In both clinical trials described above, some adverse effects appeared. In the phase II/III clinical trials, wound complications related to RT appeared equally in both treatment groups and were similar to those described in the studies of O’Sullivan et al. In the RT+NBTXR3 group, 35% of the patients demonstrated NBTXR3-related side effects. These included injection site pain (13%), grade 3–4 acute immune reaction (8%), grade 1–2 peripheral edema (9%), and hypotension (4%). However, the injection site pain was moderated with the use of analgesia and the rest of the adverse effects were manageable and short in duration.

The following table (Table 1) is a summary of the current research work for the use of NPs in RT treatment of STS.

### 3.2. The Use of Nanoparticles in Hyperthermia Treatment of Soft Tissue Sarcomas

#### 3.2.1. Liposomes and Hyperthermia

Liposomes, tiny lipid-based NPs, are increasingly recognized for their role in enhancing the effectiveness of cancer treatment when combined with HT [40,41]. By encapsulating therapeutic agents, they enable precise drug delivery to tumor sites, synergizing with HT to enable treatment directly within cancerous tissues.

Li et al., in a notable 2013 study, used mild HT to enhance liposome extravasation within tumors [42]. Liposomes are valuable vehicles for delivering chemotherapy, aiming to target tumors, while sparing healthy tissues [43]. Tumors often have heterogeneous vasculature, complicating drug distribution [44]. Murine models with different tumor types, including BFS-1 sarcoma, were employed, transplanted into dorsal skin window chambers for precise observation. The liposomes used were fluorescently labeled and contained specific phospholipids to enable accurate tracking. Mild hyperthermia at 41 °C for 1 h was applied to the tumor sites using heating coils within the chambers.

The study’s findings were highly promising. In all tumor models, including BFS-1 sarcoma, mild HT at 41 °C for 1 h substantially increased liposomal extravasation. In BFS-1 sarcoma, there was a remarkable 50-fold increase in permeability. The liposomes not only accumulated around blood vessels but also penetrated deeper into the extravascular extracellular space (EES), reaching up to 27.5 μ m(micrometers) from the vessels in certain models. Interestingly, the effect of mild HT persisted for several hours post-treatment, enhancing vascular permeability up to 8 h later, albeit to a lesser extent. Moreover, the study revealed significant heterogeneity in the extravasation process, both between different tumor models and within individual tumors.

In another study, the primary objective was to control drug release using thermo-sensitive liposomes (TSLs) [45]. The drug used was gemcitabine (dFdC), a pyrimidine analogue widely used to treat solid tumors like pancreatic, bladder, and breast cancer. However, its rapid metabolism limits its bioavailability in tumors. To tackle this, researchers developed DPPG2-TSLs loaded with dFdC. The liposomal product was characterized using dynamic light scattering (DLS) and high-performance liquid chromatography (HPLC). In vitro experiments showed increased dFdC release from these liposomes above 41 °C, with NP size impacting release rates. In vivo pharmacokinetic studies in BN175 liposarcoma-bearing rats indicated extended circulation times for larger NPs, hinting at improved drug delivery to tumors.

This animal study on BN175 tumor-bearing rats involved raising the temperature to 41 °C for one hour and then injecting TSLs. Results were compared with four other groups: saline with HT, non-liposomal with HT, non-liposomal without HT, and liposomal dFdC without hyperthermia. DPPG2-TSLs combined with HT had the most significant impact on tumor control. Importantly, this study focused on enhancing drug retention from liposomes when exposed to elevated temperatures rather than directly assessing the drug’s therapeutic efficacy. For a more comprehensive treatment approach, combining TSL-dFdC with pre- or post-delivered HT, already known to enhance dFdC’s tumor control in BN175 tumors, could be considered.

Following the first study, the same research team aimed to integrate HT to enhance vascular permeability and drug retention in a subsequent experiment [46]. They modified DOX-TSLs (thermo-sensitive liposomes) to have a higher membrane transition temperature (Tm), leading to slow drug release, termed DOX-sTSLs. The experiment used a BLM melanoma tumor model in mice and involved a two-step HT approach. Initially, they applied HT to the tumor-bearing leg, raising the temperature to 41 °C for 1 h to enhance vascular permeability (the first step HT). After a 1-minute cooling period, they systemically administered 3 mg/kg of DOX-sTSLs. Following a two-hour interval, they applied the second step HT, heating the tumor to 42 °C for 1 h.

The first step aimed to maximize DOX-sTSL accumulation in the tumor site by enhancing vascular permeability, preventing drug release into the systemic circulation. The second step aimed at achieving controlled drug release within the tumor to maximize localized cytotoxicity in vivo. In vitro survival assays were conducted using the murine BFS-1 sarcoma cell line, BLM melanoma cell lines, and HUVEC (human umbilical vein endothelial cells). Cells were exposed to 41 °C or maintained at 37 °C for 1 h in well plates. At 0, 2, and 4 h post-HT, Dox was added, and cytotoxicity was assessed after 72 h using a colorimetric XTT assay. The results showed no thermal sensitization of the cells to DOX, indicating no difference in IC50 values with and without HT. This step aimed to determine whether the positive responses observed in the subsequent animal study were due to the enhanced action of DOX in mild HT conditions or the result of improved carrier permeability and controlled drug release at the target site. Subsequent in vivo tests on BLM tumors demonstrated increased DOX release and anti-cancer efficacy after the second HT, resulting in localized drug distribution within the pre-heated tumor.

The studies described above proved the positive effect of TSLs in tumor treatment and established some liposomal formulations, suitable for targeted treatment. The HT method applied in all these studies was the thermal bath, where the tumor bearing part is placed in water.

In a related study, researchers examined the influence of different HT methods, such as cold-light and diode 940 nm laser-light application systems, in combination with TSLs for drug delivery [47]. They also investigated the impact of tumor size on treatment outcomes. Using the BN175 tumor model, tumors were cultivated in culture medium and later injected into the hind legs of mice. Tumors were allowed to grow to specific sizes, dividing the mice into small-tumor and large-tumor groups. The treatment involved maintaining the tumor sites at a stable 40 °C for one hour, followed by intravenous injection of liposomes containing DOX. Subsequently, HT was applied for 60 min to reach a minimum intratumoral temperature of 40 °C. The study revealed effective DOX delivery, with higher concentrations in heated tumors compared to non-heated ones. Additionally, smaller tumors displayed higher DOX levels. Interestingly, the laser and cold-light delivery methods demonstrated superior DOX concentrations compared to the water bath approach, possibly due to the former methods’ ability to generate higher temperature gradients.

Peller et al. explored the magnetic resonance (MR) compatibility of a laser applicator and the visualization of HT-induced drug release [48]. A contrast agent (Gd-DTPA-BMA) was encapsulated in liposomes instead of DOX to induce signal enhancement after release in the tumor. In this sub-experiment, a mouse bearing small BN175 tumors was imaged using a 3T clinical MRI system with dynamic imaging. Following liposome injection, rapid signal increase was observed in both tumors within the first 30 s. However, only the signal from the heated tumor continued to rise afterward, reaching 1.7-times the baseline signal before injection. In contrast, the signal from the non-heated tumor remained constant after the initial increase, reaching only 0.38-times the baseline signal. These images indicated a significant release of the contrast agent inside the laser-heated tumor compared to the non-heated one.

The MRI experiment described above aimed to observe targeted drug release within the tumoral environment. However, MRI can serve another crucial role in HT treatment. One of the critical challenges in HT is the uneven distribution of temperature within tumors, making it difficult to precisely monitor and control. To address this issue, researchers have harnessed MRI combined with TSLs to offer a more accurate mapping of the temperature changes induced by HT [48].

The principle behind this approach is that when the temperature surpasses a specific threshold for TSLs, they undergo a transformation allowing water exchange through their membranes, releasing a contrast agent (CA). This CA release influences the MRI signal, serving as a marker for temperature increases within the tumor. Additionally, the movement of CA from the bloodstream to the interstitial space creates a washout effect, linked to drug distribution. In the study conducted by Peller et al., CA-loaded TSLs were integrated with TSLs containing the anti-cancer drug DOX, to overcome potential limitations related to drug dosing.

For the purposes of this study, rats with BN175 tumors were divided into two groups: one receiving HT treatment (Group A) and the other serving as a control (Group B). The HT protocol involved elevating the tumor temperature to a target of 40 °C, followed by the intravenous injection of a combination of CA-TSLs and DOX-TSLs. Subsequently, HT was applied to the tumors for a duration of 60 min. After a cooling period, the rats were euthanized, and both the treated and untreated tumors were surgically removed for analysis.

The results of this study showcased a significant increase in drug concentration within the HT-treated tumors compared to the untreated ones. Moreover, there was a clear positive correlation between temperature levels and drug release. In addition to evaluating drug concentration, the researchers examined various MRI parameters to monitor temperature changes. While these parameters provided valuable information up to the point of injection, they exhibited limitations in tracking temperature variations during the treatment phase. This led to the conclusion that one specific parameter, phase contrast PC(t), was better suited as a marker of DOX concentration rather than as a reliable tool for real-time thermometry during treatment.

An alternative approach using temperature-dependent hydrogels has also been investigated [49]. Hydrogels are hydrophilic polymers that remain insoluble in water within a specific temperature range known as the lower critical solution temperature (LCST) [50]. For in vivo thermally induced drug release, the hydrogel’s LCST should ideally fall between 37 °C and 43 °C. In this study, the primary monomer for the hydrogel was the amphipathic molecule NIPA (N-isopropylacrylamide). The resulting poly(NIPA-co-AAm) nano-hydrogel (NHG) was produced through free-radical precipitation polymerization, with a particle size of 50 nm. The LCST was determined as 40 °C by monitoring NHG’s optical transmittance in a water bath, where it transitioned from transparent to opaque.

After characterizing NHG and assessing its drug loading capacity with model drugs, including docetaxel (DTX), an in vitro drug release profile was established. Subsequently, an in vivo therapeutic experiment was conducted using mice bearing S180 murine sarcomas. The mice were divided into four groups: a control group receiving saline, a group with free DTX injection, a group with DTX-NHG without HT, and a group with DTX-NHG combined with HT using a water–sack system. DTX-loaded NHG with exhibited the highest tumor growth inhibition rate, with minimal systemic cytotoxicity. This approach supported the mice’s overall well-being and demonstrated the potential of hydrogels for controlled drug release in HT-based cancer treatments.

#### 3.2.2. Liposomes for Combined Hyperthermia and Radiotherapy

A 2018 study proposed an innovative approach for enhancing radiation therapy’s (RT) effectiveness while minimizing side effects by combining it with thermo-sensitive chemotherapeutic agents [51]. RT often faces limitations due to the risk of radiation-induced toxicity in healthy surrounding tissue, despite its potential for effective tumor control. Combining chemotherapy with RT, known as chemo-radiation, is a standardized approach but often leads to severe side effects. This study aimed to address these issues by utilizing hyperthermia to achieve targeted, local drug release alongside RT.

The study used the HT1080 fibrosarcoma cell line and tested various combinations of TSL temperatures (37 °C to 43 °C), single doses of X-ray radiation (0 to 8 Gy) from a linear accelerator, and chemotherapy using free doxorubicin (DOX) or thermoDOX (TSLs encapsulating DOX). Cell survival assays were conducted on day 7, and flow cytometry analyzed phosphorylated-H2AX staining to assess double-strand breaks at 45 min and 24 h post-RT. ThermoDOX had minimal effects at lower temperatures (37 °C), indicating DOX encapsulation prevented release without HT. However, at higher temperatures, thermoDOX exhibited similar cytotoxicity to free DOX. Furthermore, combining HT and chemotherapy improved the sensitivity to radiation, before or after RT, suggesting potential effectiveness for tumor control, while minimizing side effects.

#### 3.2.3. Magnetic Nanoparticles and Hyperthermia

Magnetic nanoparticles (MNPs) are another approach using NPs, which can be used to induce heat through an alternating magnetic field, directing them to tumor sites with an external magnetic field. This method is called magnetic nanoparticle hyperthermia (MNH). MNPs absorb energy from alternating magnetic fields, converting it into heat, raising the temperature of cancer cells [52]. Precisely controlling this heat generation allows localized HT, effectively damaging cancer cells, while sparing healthy tissue. This approach seems promising for cancer patients, as a minimally invasive and targeted treatment. However, MNH faces limitations, including the risk of exceeding a critical temperature threshold (46 °C), leading to severe damage to healthy tissues.

Researchers have enhanced temperature monitoring during MNH by combining infrared thermography (IRT) with numerical simulations [53]. This method creates a thermal map of the treatment surface, with each voxel indicating the temperature measured by an individual thermometer. To mitigate errors, a mathematical thermography error model was incorporated. For their study, researchers utilized the S180 murine tumor model and manganese-ferrite NPs. The results revealed that IRT measurements strongly depended on the measurement angle, indicating temperature underestimation in previous MNH studies. The alignment between simulation and animal data suggests that integrating this model with IRT could enable precise temperature monitoring during MNH, with implications for dosimetry and treatment planning.

Self-controlled HT in MNH relies on the Curie point, a temperature where magnetic materials undergo significant changes in their magnetic properties. MNPs with Curie points between 42 and 46 °C can terminate heat generation, providing a means of precise temperature control.

In one study, magnetic liposomes were developed for self-controlled HT and chemotherapy [54]. These liposomes contained paclitaxel, a potent anti-tumor drug, coated with dextran for controlled release. In vitro tests on L929 cells showed improved biocompatibility with a higher Fe_3_O_4_ to LSMO ratio and dextran coating. Cytotoxicity studies on HT-180 cells demonstrated reduced cell viability when paclitaxel was encapsulated in the MNPs, further enhanced by HT.

In vivo biocompatibility tests on liver and kidney function revealed no significant abnormalities. Therapeutic evaluation of magnetic liposomes with HT showed tumor response, with minimal MNPs presence in other organs. These results indicate the potential of MNPs for in vivo treatment, warranting further investigation

Table 2 contains the main research work conducted on the implementation of NPs in HT treatment of STS.

## 4. Discussion

The primary objective of this paper was to conduct a comprehensive review of the existing literature concerning the treatment of STS using NPs in RT and HT treatment protocols.

In nearly all of the studies examined, the utilization of NPs demonstrated significant anti-cancer activity. The majority of these investigations, as presented in this review, relied on in vitro experiments and in vivo assays involving murine models. These experiments primarily focused on aspects such as NP uptake by cells, cytotoxicity assessments, and survival tests using STS cell lines in vitro. Except for the phase I and phase II/III clinical trials involving NBTXR3, most of the research presented here was based on in vivo animal studies. NBTXR3 is a promising radiosensitizer for radioresistant soft tissue sarcomas, showing statistically significant improvements over radiotherapy alone. This suggests its potential to enhance prognosis and outcomes for patients with these challenging malignancies, with further research needed to understand grade-specific responses.

As mentioned earlier, in the phase II/III clinical trial conducted by Bonvalot et al. the randomization of the sample was stratified by histological subtype and myxoid liposarcoma vs. other types. Myxoid liposarcoma is a STS subtype that exhibits high radiosensitivity [56]. The stratification was conducted to ensure that each treatment group had a balanced representation of patients with liposarcoma vs. other subtypes. This method led to smaller errors and greater precision than simple random ranking [57]. Furthermore, in that study, patients with STS that were localized in the anterior abdominal region and tumors whose volume exceeded 3000 mL at baseline were excluded. This was undertaken because those tumors needed administration of more than 300 mL of NBTXR3, which was not feasible. Such tumors are retroperitoneal STS, which usually remain undiagnosed for long periods of time and grow large inside the abdominal cavity. Only half of these tumors can be completely removed through surgery and they demonstrate a 90% recurrence rate [58].

In the case of HT, liposomes seem to be a promising avenue for more efficient and targeted cancer therapies, making them a pivotal component in advancing hyperthermia-based treatments. Overall, the studies included in this review demonstrated the potential of combining mild hyperthermia with liposomes to significantly increase drug extravasation in tumor tissues, thus enhancing chemotherapy efficacy. However, the success of this approach varied depending on the tumor characteristics, such as vascular density and the structure of endothelial linings, as described by Li et al. [42]. These findings underscore the complexity of tumor biology and highlight the need for further research to harness the full potential of this promising therapeutic strategy.

The lack of human trials for STS treatment aligns with the broader pattern in cancer research. Only a few such trials have been reported in the literature for RT, including a phase I/II clinical trial involving hafnium oxide NPs activated by SABR for advanced HNSCC treatment [59]. For HT, the current literature includes a few clinical trials, regarding MHT for the treatment of brain tumors in combination with RT [15] and thermal ablation of prostate cancer [60]. Nevertheless, numerous ongoing clinical trials and discontinued trials have been documented.

Considering the promising outcomes of STS experiments so far, it is reasonable to anticipate the progression of preclinical trials. Given the demonstrated effectiveness of NPs in various applications discussed in this project, their potential should be further explored in human patients. As previously discussed, STS are aggressive tumors, and the development of novel complementary treatment modalities is crucial, especially for younger patients.

However, despite the encouraging results of the studies described in this review, certain issues need to be addressed regarding the use of NPs. Most of the in vivo murine studies were conducted using xenografted tumors, which may be useful as indicators of treatment potential but do not fully replicate the complexity of real tumor environments. Real tumors present challenges related to vasculature, homogeneity, and overall anatomy that may affect NP retention. Additionally, the growth pattern of spontaneous tumors may interfere with neighboring tissues and organs, leading to challenges in achieving therapeutic margins with RT or hyperthermia. Consequently, the use of genetically engineered murine models may offer a more representative approach to cancer treatment [61].

The studies featuring murine cancer models or xenografted tumors in mice had as a primary objective confirmation of the successful retention of NPs at the tumor site. Only a limited number of studies aimed to determine the pharmacokinetic profile of NPs and identify optimal dosages. It is important to note that these experiments were not intended to develop the most effective treatment regimen but rather to establish the potential enhancement of common treatment protocols through NP utilization. For that reason, it is not safe to draw conclusions regarding treatment parameters such as optimal NP diameter, concentration, and time gap between injection and treatment in RT and HT, and optimal temperature and heating time in HT. These parameters should be further assessed in the future.

NPs can be administered either intravenously or intratumoraly. Intratumoral injection seems to have a better distribution of NPS, offering a tremendous enhancement in RT dose in the gross tumor volume [62], whereas intravenous administration seems to have inferior upload inside the cancerous tissue, although organic NPs such as liposomes seem to have sufficient tropism for the solid tumor [63]. In terms of clinical practice, it is important to consider that NPs may need to be administered during each treatment session. This not only raises concerns about the administration route and potential toxicity but also poses questions about the overall cost of treatment. Moreover, the reproducibility of NP distribution is limited, meaning that the final target of each treatment session may differ from previous ones [64,65]. Consequently, treatment planning may need to be patient- and fraction-specific to optimize outcomes and minimize potential side effects. Furthermore, the implementation of NPs may not be always easy to achieve within the current routines of cancer treatment departments.

Apart from in vitro and murine in vivo studies, a few veterinary studies involving cats with FISS treated with NP-enhanced RT [29] and HT [30] and a single case of STS in a canine patient treated with NP-enhanced RT [31] have also been conducted. Although these treatments appeared successful, the sample sizes were limited. Particularly in the case of the dog sarcoma, the study relied on one veterinary patient, lacking reproducibility, statistical analysis, or comparisons. It is worth noting that the development of veterinary treatments is not unrelated to human treatments. Key aspects of treatment methods, such as radiation delivery and drug administration, share similarities between veterinary and human medicine. Companion animals in particular can make a unique contribution to the improvement of radiation oncology practice [66]. However, achieving interspecies translatability is not always guaranteed, leading to instances of “translational failure” [67].

It is generally accepted that NPs are a novel tool for safer drug administration and increased therapeutic efficacy. However, some safety concerns remain under research. The physicochemical characteristics of nanoparticles, including particle size, morphological and structural variations, surface charge, surface chemical modifications, chemical properties, composition, aggregation state, and stability, play crucial roles in influencing their biosafety. Additionally, factors such as concentration, crystallization, route of administration, dose, tissue distribution range of nanoparticles, cell type, and animal species are all contributors to their biosafety [68]. These variables impact cellular uptake, intracellular transport and localization, interaction with biomolecules like proteins and DNA, as well as in vivo distribution and metabolic clearance, thereby causing specific biological effects.

Gold is thought to be generally safe; however, the surface of GNPs is chemically active, meaning they can interact with blood proteins and membrane surfaces. In the study of Joh et al. [33] described earlier, the deposition of the GNPs after intravenous injection was 10- and 80-times higher inside the tumor than in muscle and brain, respectively, but there was still some deposition outside the tumor, which was not monitored in terms of adverse effects. Pharmacokinetics of GNPs are not well documented in humans. Comparably, NBTXR3 presents a good overall intratumoral dispersion, but inductively coupled plasma mass spectrometry demonstrated a leakage of less than 10%. However, their physicochemical state and crystalline structure make them a safer option [39].

In a similar manner, liposomes are used for reduction of the systemic toxicity that otherwise is caused by their encapsulated agents. However, the liposomes themselves can induce toxicity to normal tissues. For instance, DOX is known to be accumulated in the liver and spleen due to the action of the reticuloendothelial system (RES), causing severe adverse effects [69]. PEG is used to reduce the systemic toxicity and enhance the circulation time of the liposomal drugs by reducing the blood protein absorption, but a similar impact on liver function has been documented.

For MNPs, the main concerns include the inflammation caused after migration to neighboring organs, and the intracellular accumulation of metals after the physiological degradation of the MNPs [70].

Another critical consideration for the use of NPs is the potential for long-term side effects. For instance, while GNPs are generally considered non-toxic and biocompatible, a study published in 2016 revealed that low-dose exposure to GNPs could induce lasting changes in human cells, with the effects varying based on the type of NP and cell involved. Both acute and prolonged exposure were found to alter gene expression related to the cell cycle, resulting in morphological changes and cellular stress in fibroblasts [71]. As previously discussed in this paper, fibrosarcoma is one of the tumor models treated with NPs. The induction of oxidative stress and inflammation in fibroblasts raises concerns, as this may disrupt the homeostasis of the surrounding connective tissue cells. Overall, the interaction of NPs with radiation has been well documented; however, it is not yet clear how different agents may interfere with the tumor micro-environment and how they may affect tumor immunogeneity [72].

In the case of NPs used for MNH, the NPs must also respond to specific criteria, like heat dose production, magnetoenergy conversion, and surface chemistry [73]. However, magnetic nanoparticle hyperthermia (HT) faces limitations, including the risk of exceeding a critical temperature threshold (46 °C), leading to severe damage to healthy tissues. Monitoring temperature changes during magnetic nanoparticle hyperthermia presents challenges. MRI is impractical due to interference with the magnetic field, and alternative methods introduce significant errors. Achieving accurate temperature measurements in tumors requires numerous thermometers, which is infeasible in in vivo treatment. Thus, it is important for new temperature monitoring techniques to be developed like the one implemented by Rodrigues et al. [53].

## 5. Future Perspectives

In the ever-evolving landscape of soft tissue sarcoma (STS) treatment, future perspectives hold immense promise, particularly regarding the integration of HT and RT with cutting-edge NP-based approaches. These novel strategies have the potential to revolutionize the way we combat STS, offering more precise, effective, and personalized treatment options.

One exciting avenue of exploration involves the utilization of MRI-compatible Gd-NPs. These NPs not only offer post-treatment imaging capabilities but could also play a pivotal role during the treatment process itself. With the advent of MR-LINAC devices, which seamlessly integrate MRI into RT sessions [74], Gd-NPs could enable real-time MRI imaging during RT. This advancement promises to enhance treatment precision and monitoring, ultimately improving patient outcomes. This combination has already been implemented in a phase I clinical trial for MRI image-guided WBRT of brain metastasis with novel AGuIX (activation and guidance irradiation X) gadolinium nanoparticles [75], and there is a pending phase II trial.

Boron neutron capture therapy (BNCT) represents another promising approach, since it has been previously used for radioresistent tumors like STS [76]. By irradiating nonradioactive boron-10 with low thermal neutrons, BNCT generates alpha particles with a high linear energy transfer (LET), depositing their energy with remarkable precision [77]. Interestingly, Gd-NPs exhibit a neutron capture cross-section 60-times greater than that of Boron. This revelation opens the doors to gadolinium neutron capture therapy (GdNCT), a potentially groundbreaking treatment modality for STS. Developing effective Gd-carrying agents for GdNCT, such as nanocomposites and solid-state Gd-NPs, not only holds the potential to improve cancer-killing rates but would also enable MRI-guided neutron capture therapy (NCT), further enhancing treatment accuracy [78]. A non-randomized phase II trial is currently recruiting for hypofractionated proton therapy with the use of nano-chelates of gadolinium and polysiloxane [79].

The significance of NPs lies in their versatility for multimodal treatments, as illustrated in this paper. Immunotherapy is a treatment method that has shown promise in STS treatment. Another avenue in immunotherapy involves its synergistic action with RT and hyperthermia HT. Both RT and HT can induce an anti-cancer immune response at the irradiation site by promoting the presentation of tumoral neoantigens to T lymphocytes. However, this immune response may not be potent enough to have a therapeutic impact. The use of RT/HT in combination with NPs modified for chemo-immunotherapy holds promise [80]. A phase I/II randomized trial with the use of NBTXR3 is currently recruiting in order to assess the abscopal effect of lung and liver solid tumor metastasis [81]. Additionally, the genomic landscape seems important for precision medicine, while gadolinium oxide nanoparticles with low-dose RT have shown interesting results for chondrosarcoma spheroids in terms of an in vivo mouse model [82].

Furthermore, the use of brachytherapy in STS treatment should be considered. As mentioned earlier, external beam radiation therapy (EBRT) presents challenges in avoiding the irradiation of healthy tissues. Therefore, brachytherapy is employed as a highly precise treatment technique, positioning applicators precisely at the desired location [83]. The tumor is irradiated directly by an internal source, with the radiation being attenuated before reaching the healthy surrounding tissues. However, brachytherapy has limitations, including operational difficulties, patient discomfort, and post-treatment side effects. Research is exploring the use of radio-NPs for brachytherapy, as an alternative to conventional radioactive seeds. Clinical studies on localized delivery of radio-NPs, especially with radiolabeled GNPs, have been reported [84]. In the future, these NPs could be employed in the brachytherapy treatment of STS [85].

## 6. Conclusions

In conclusion, this comprehensive review underscores the immense potential of NPs for addressing the formidable challenges in STS treatment, including radio-resistance and aggressive metastasis. NPs have shown great potential for enhancing the effectiveness of RT and HT, offering targeted treatment options through radio sensitization, TSLs, and MNPs. While preclinical studies have demonstrated their promise, the limited clinical trials, with only a phase I and a phase II/III trial for RT using hafnium oxide NPs, highlight the need for broader clinical implementation in the future, holding the potential to revolutionize STS therapy and significantly improve patient outcomes. Thus, more clinical trials are needed for the extraction of safe results and consequently the further implementation of hyperthermia combined with nanoparticles in routine clinical practice.

## Figures and Tables

**Table 1:** Studies with NPs for STS radioenhancement.

	NPs Type	Sarcoma Type	Study Type	Aim	Outcome	Reference
GNPs	P-GNPs	HT1080 fibrosarcoma	in vivo	Imaging of NP’s accumulation and irradiation results	Extended NP’s blood circulation, regression of tumor growth	[33]
	Gold nanospheres	Feline Injection Site Sarcoma (FISS) cells	in vitro	Cytotoxicity assay	Decreased viability of cells	[29]
	RGD-GNPs, PEG-GNPs	Mouse primary STS	in vitro/in vivo	Assess of vascular permeability enhancement post RT and enhanced chemo-drug ability	Lower vascular permeability threshold, enhanced chemo-drug intratumorally delivered	[35]
Hafnium oxide NPs	NBTXR3	HT1080 fibrosarcoma	in vitro/in vivo	Tumor growth and cytotoxicity assay	Significant enhancement of RT outcomes	[39]
		STS of the extremities or the abdomen	Phase I	Determination of Maximum Tolerated Dose (MTD) and pathological response, 22 patients	Optimal dose set as 55.3 g/L of NBTXR3 at a volume equivalent to 10% of the calculated baseline tumor volume	[27]
			Phase II/III clinical trials	Evaluate effectiveness and side effects, 180 patients	Increased anti-tumor efficacy and tumor response than RT alone	[28]
Folic calcium tungstate NPs	FOL-PEG-PLA/CWO	Canine STS	Veterinary	Evaluate effectiveness and side effects	Translatable use of NPs from murine models, significant tumor shrinkage	[31]

**Table 2:** Studies with NPs for STS treated with hyperthermia.

	NP Type	STS Subtype	Study Type	Aim	Outcome	Reference
Liposomal NPs	DPPC/DSPC/DS PE-PEG200	BFS-1	in vivo	Assessment of vascular permeability with window chambers	fifty-fold increase in permeability	[42]
	DPPG2-TSLs loaded with dFdC	BN175	in vivo	Controlled drug release with thermosensitive liposomes	Enhanced drug retention with temperature rise, enhanced tumor control	[45]
	DOX-TSL	BFS-1	in vitro	Complementary survival assay of two-step hyperthermia	No thermal sensitization of cells to DOX alone	[46]
		BN-175	in vivo	Method of hyperthermia and tumor size influence on TSL treatment	Laser and cold-light more effective than water bath, greater concentration of DOX in small tumors	[47]
	CA-TSL & DOX-TSL	BN-175	in vivo	Determination of MRI markers for temperature and drug release monitoring	No significant results for temperature monitoring, PC(t) marker for in vivo chemo-drug dosimetry	[48]
	DPPG2-TSL-DOX	FISS	Veterinary	Pharmacokinetic analysis and tumor response	Rapid elimination from the circulation and increased extravascular release	[30]
	ThermoDox	HT180	in vitro	Combination of HT and chemo drugs to enhance RT treatment	Same radio-enhancement results when HT+chemo was applied either pre or post RT	[51]
	DTX-NHG	S180	in vivo	Anti-tumor efficacy of DTX-NHG vs DTX	Higher inhibition rate and fewer side effects of DTX-NHG	[49]
Magnetic NPs	manganese ferrite DMSA	S180	in vivo	Development of novel infrared thermography technique for HT	Successful Integration of IRT with numerical simulations	[55]
	LSMO+Fe_3_O_4_	HT1080	in vivo	Cytotoxicity and therapeutic evaluation tests of NPs for Chemo+self-controlled HT	Positive tumor response, no significant presence of the NPs outside the tumor	[54]

## Abbreviations

The following abbreviations are used in this manuscript:

STS	Soft Tissue Sarcoma
RT	Radiotherapy
HT	Hyperthermia
NP	Nanoparticle
GNP	Gold Nanoparticle
p-GNP	Pegylated Gold Nanoparticle
RD	Recommended Dose
pCR	Pathological Complete Response
DOX	Doxorubicin
TSL	Thermosensitive Liposomes
CA	Contrast Agent
LCST	Lower Critical Solution Temperature
NHG	Nanohydrogel
MNH	Magnetic Nanoparticle Hyperthermia
IRT	Infrared Thermography
